# Laparoscopic Right Colectomy with Intracorporeal Handsewn Anastomosis: Surgical Technique and Narrative Review of Literature

**DOI:** 10.3390/medicina62030551

**Published:** 2026-03-16

**Authors:** Theodora Choratta, Konstantina Spyridaki, Dimitra Ntrikou, Michael Lazaris, Melina Papalexandraki, Lazaros Kourtidis, Katerina Neokleous, Marilena Tsivgouli, Athanasios Kalligas, Efstratios Kouroumpas, Dimitrios Margaritis, Panagiotis Dikeakos, Christos Iordanou, Georgios Ayiomamitis

**Affiliations:** Laparoscopic Unit, 1st Surgical Department, General Hospital of Piraeus “Tzaneio”, 18536 Piraeus, Greece; theodora.choratta@yahoo.com (T.C.); konstantinasp.gr@gmail.com (K.S.); dimitra.ntrikou@gmail.com (D.N.); mixlazar@gmail.com (M.L.); melinapapalex@gmail.com (M.P.); l.kourtidis@gmail.com (L.K.); katerneo1995@gmail.com (K.N.); mtsivgouli@gmail.com (M.T.); thkalligas@me.com (A.K.); ekouroumpas@gmail.com (E.K.); d.mrg@hotmail.com (D.M.); dikeakosp@gmail.com (P.D.); chriordanou@yahoo.gr (C.I.)

**Keywords:** laparoscopic right colectomy, intracorporeal anastomosis, handsewn anastomosis, minimally invasive colorectal surgery, surgical technique

## Abstract

Intracorporeal anastomosis (IA) has gained increasing acceptance in minimally invasive colorectal surgery, primarily owing to its demonstrated association with improved perioperative outcomes compared with extracorporeal techniques. Nevertheless, the specific role of intracorporeal handsewn anastomosis remains insufficiently explored within the context of laparoscopic colorectal procedures. The present study describes a standardized technique for performing a side-to-side isoperistaltic handsewn intracorporeal ileocolic anastomosis following laparoscopic right colectomy and evaluates its safety and feasibility through a review of the relevant literature and institutional experience. The procedure is executed employing a medial-to-lateral dissection approach, and a single-layer isoperistaltic handsewn anastomosis is constructed entirely intracorporeally. Over a three-year period, 68 laparoscopic right colectomies were completed using this technique, predominantly for malignant disease, all performed by a single surgeon. Notably, no anastomotic leaks or anastomosis-related complications, including bleeding, stenosis, or hematoma formation, were observed. Available evidence supports the advantages of intracorporeal anastomosis, including reduced surgical trauma, lower incidence of wound-related complications, faster recovery of bowel function, and comparable oncological outcomes. Furthermore, emerging data from robotic-assisted colorectal surgery suggest potential benefits of handsewn techniques with respect to hemostasis and anastomotic quality. In conclusion, intracorporeal handsewn ileocolic anastomosis following laparoscopic right colectomy appears to represent a safe and reproducible technique when performed by experienced surgeons, thereby warranting further prospective, comparative and multicenter studies to delineate its broader applicability and long-term outcomes.

## 1. Introduction

Since the pioneering reports of laparoscopic-assisted colectomies published independently by Jacobs et al. [1] and Fowler and White [2] in 1991, laparoscopic colorectal surgery has undergone substantial evolution and widespread adoption across surgical practice worldwide. Over the ensuing decades, this minimally invasive approach has consistently demonstrated several well-established advantages over conventional open surgery, including reduced postoperative pain with diminished opioid analgesic requirements, lower rates of surgical site infection, decreased incidence of incisional hernia formation, and an overall improvement in postoperative recovery [3,4,5]. These benefits have been further augmented with the introduction of totally laparoscopic procedures incorporating intracorporeal anastomosis. The earliest reports of intracorporeal anastomosis in the context of colorectal surgery date back to 1992, with subsequent detailed descriptions of the technique specifically applied to laparoscopic right hemicolectomy provided by Casciola et al. [6] in 2003 and Lechaux [7] in 2005.

Despite these considerable advances, a persistent and ongoing debate continues regarding the comparative merits of intracorporeal (IA) versus extracorporeal anastomosis (EA) in minimally invasive colorectal surgery, which has contributed to the relatively limited adoption of totally laparoscopic techniques. Over the past two decades, multiple studies—including randomized controlled trials, systematic reviews, and meta-analyses—have demonstrated comparable, and in many instances superior, outcomes associated with intracorporeal anastomosis when compared to extracorporeal approaches [8,9,10,11,12]. These investigations have primarily focused on evaluating the safety and efficacy of laparoscopic intracorporeal anastomosis, particularly in the setting of malignant disease, while concurrently assessing oncologic adequacy and radicality of resection.

The principal concern associated with intracorporeal anastomosis pertains to its safety profile, with anastomotic leakage representing the most clinically significant and potentially devastating manifestation of anastomotic failure. Reported rates of anastomotic leakage following laparoscopic right colectomy in international literature range from approximately 1% to 8%, although a notable declining trend has been observed in more recent series [10,13,14]. Several factors are recognized to influence anastomotic healing and are commonly categorized into modifiable and non-modifiable preoperative factors, tumor-related factors, and intraoperative risk factors [13]. Among modifiable risk factors, nutritional status, obesity, immunosuppression, and neoadjuvant therapy have been identified as significant contributors to anastomotic failure [14].

Fundamental surgical principles identify three key technical determinants of successful anastomosis: (1) meticulous surgical technique with careful prevention of hematoma formation; (2) preservation of adequate blood supply to the bowel ends to prevent ischemia during dissection; and (3) the creation of a tension-free anastomosis [15,16,17,18,19]. When these principles are rigorously and consistently applied during the performance of intracorporeal anastomosis, the risk of anastomotic failure can be maintained at acceptably low levels. 

At present, the majority of surgeons performing intracorporeal anastomosis employ mechanical devices such as linear or circular staplers, selected according to the type of anastomosis and the specific anatomical requirements of the procedure. In the context of laparoscopic right colectomies, linear staplers represent the most commonly utilized devices owing to their convenience, versatility, and adaptability, particularly with the advent of technologically advanced stapling platforms offering articulation angles of up to 110 degrees. Although intracorporeal handsewn anastomosis constitutes a cost-effective alternative that eliminates the need for expensive disposable stapling devices, it necessitates advanced laparoscopic suturing skills and is associated with a steeper learning curve. Furthermore, handsewn anastomosis is generally more time-consuming to construct. Consequently, owing to its considerable technical demands and training requirements, this approach has been less frequently adopted in clinical practice, and there exists a paucity of literature evaluating its outcomes in comparison with stapled intracorporeal anastomosis, particularly in the laparoscopic setting.

The aim of the present study is to describe in detail the standardized intracorporeal handsewn anastomotic technique that has been systematically implemented in our surgical unit following laparoscopic right colectomy, and to evaluate its safety and efficacy through a narrative review of the relevant literature.

## 2. Materials and Methods

### 2.1. Literature Search

A narrative review of the current literature was conducted using the PubMed/MEDLINE and Scopus databases. The search strategy included the following terms, alone and in combination: “intracorporeal anastomosis,” “extracorporeal anastomosis,” “handsewn anastomosis,” “stapled anastomosis,” “laparoscopic right colectomy,” “right colectomy,” and “ileocolic anastomosis.” No date restrictions were applied. Original articles, randomized controlled trials, systematic reviews, meta-analyses, and narrative reviews published in the English language were considered. Reference lists of retrieved articles were also screened to identify additional relevant studies. The literature was critically appraised and synthesized in the context of the authors’ clinical experience with intracorporeal handsewn ileocolic anastomosis.

### 2.2. Surgical Technique

Optimal positioning of the patient, surgical instruments, and the operating team is of paramount importance to ensure precise tissue dissection and the construction of a tension-free anastomosis. Proper ergonomics not only enhance surgical accuracy, but also reduce fatigue and facilitate advanced intracorporeal maneuvers, particularly suturing. Patients are positioned in the modified Lloyd-Davies position, with a gentle left-sided reverse Trendelenburg tilt, thereby facilitating gravitational displacement of the abdominal viscera toward the left and maximizing exposure of the right colon and its mesenteric attachments.

The primary surgeon assumes a position between the patient’s legs, ensuring a central and ergonomic operating position. The first assistant is positioned on the patient’s left side, primarily responsible for camera handling and exposure, while the second assistant stands on the right side to facilitate retraction and maintain optimal tissue tension. This arrangement promotes coordinated movement and effective instrument triangulation throughout both the dissection and reconstructive phases of the procedure.

**Figure 1:**
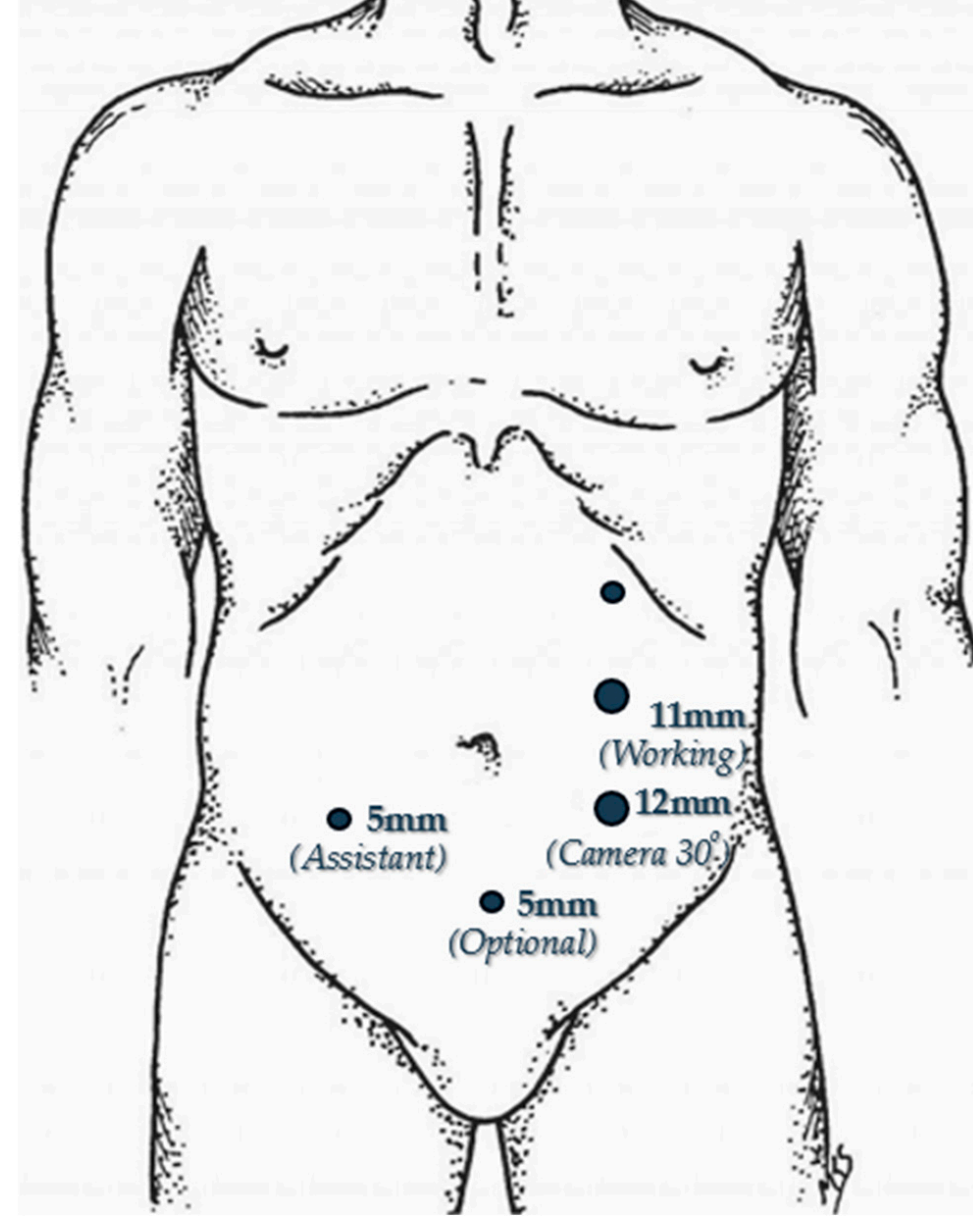
Trocar placement.

Pneumoperitoneum is established at a pressure of 12–15 mmHg utilizing a 5-mm optical trocar inserted in the left upper quadrant at Palmer’s point, situated below the left costal margin along the anterior axillary line. Following adequate insufflation and initial inspection, the working trocars are positioned as follows: an 11-mm trocar in the left upper quadrant along the midline between the umbilicus and Palmer’s point; a 12-mm trocar in the midline between the umbilicus and the left anterior superior iliac spine; and an additional 5-mm trocar in the right lower quadrant, positioned along the midline between the umbilicus and the right anterior superior iliac spine, serving primarily for retraction assistance. An additional 5 mm trocar may be placed in the suprapubic region to facilitate retraction when required (Figure 1). This trocar configuration provides optimal triangulation for both the dissection and the subsequent intracorporeal suturing phases of the procedure.

The procedure commences with a systematic assessment of the peritoneal cavity in order to exclude the presence of peritoneal dissemination or carcinomatosis. In the event that suspicious secondary lesions are identified, targeted biopsies are obtained for histopathological evaluation. The colonic lesion is subsequently identified and carefully assessed to determine its precise location, extent, and relationship to adjacent structures, thereby defining the appropriate extent of resection.

**Figure 2:**
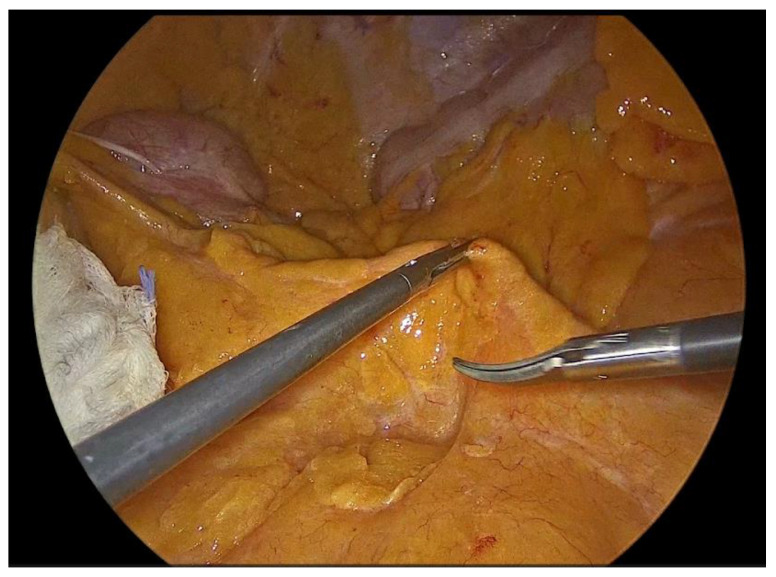
Identification of the Ileocolic pedicle.

**Figure 3:**
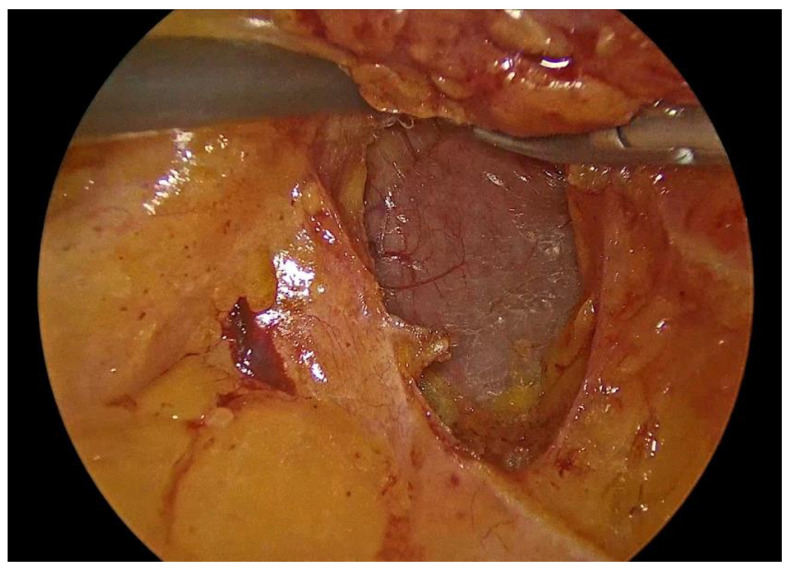
Incision of the mesenteric peritoneum and development of the avascular plane to the retroperitoneum with protection of the duodenum.

**Figure 4:**
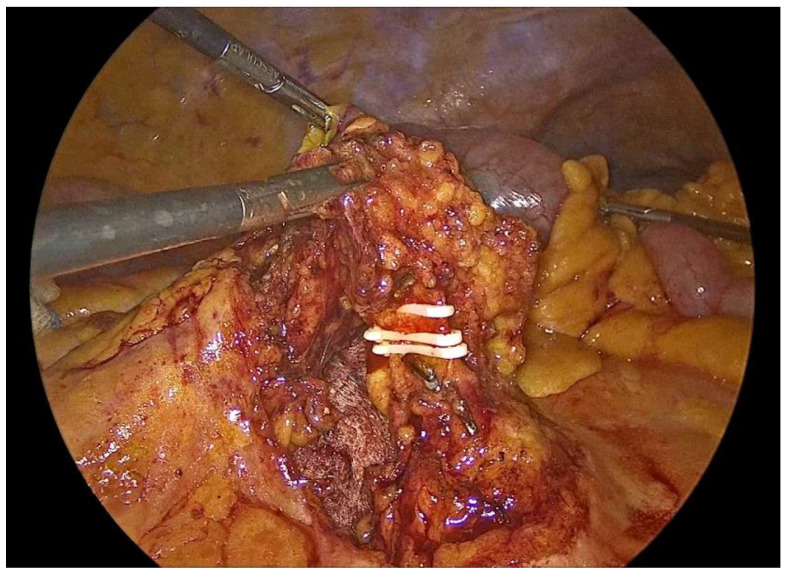
Transection of the ileocolic vessels.

**Figure 5:**
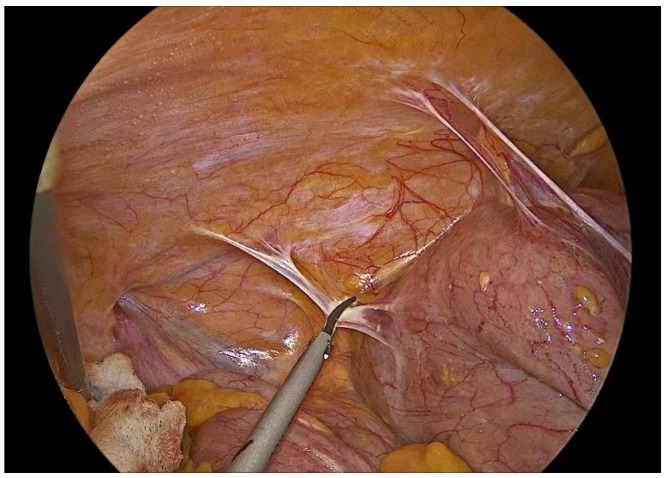
Lateral mobilization of the ascending colon.

Dissection is performed utilizing a medial-to-lateral approach. The ileocecal junction is first identified, followed by exposure of the ileocolic vascular pedicle (Figure 2). The mesenteric peritoneum is incised using an ultrasonic energy device, and the avascular plane between the mesocolon and the retroperitoneum is developed through a combination of blunt and sharp dissection, with meticulous attention paid to preserving the integrity of the retroperitoneal structures. Dissection proceeds laterally along the embryological plane, following Toldt’s fascia, while carefully identifying and protecting the duodenum, right ureter, and gonadal vessels (Figure 3). Once the dissection along this plane is completed, the ileocolic vessels are divided between polymer locking clips (Hem-o-lok) at their origin (Figure 4), just inferior to and below the duodenum, thereby ensuring complete excision in accordance with oncologic principles. Cranially, the dissection follows the superior mesenteric vein up to the middle colic vein, where the right branches of the middle colic vessels are divided between polymer locking clips. The dissection then proceeds from above through the gastrocolic ligament with the patient repositioned in the reverse Trendelenburg position. Care must be taken to identify the correct dissection plane through the lesser sac while maintaining the integrity of the mesogastrium. Subsequently, the dissection follows the transverse colon toward the hepatic flexure and continues caudally toward the cecum, while the ascending colon is mobilized from its lateral peritoneal attachments (Figure 5).

Following complete mobilization of the specimen and transection of the mesocolon and its associated vasculature, the terminal ileum and transverse colon are divided using a 60-mm endoscopic linear stapler. The specimen is temporarily repositioned over the liver to maximize the operative field and facilitate the unobstructed construction of the anastomosis.

**Figure 6:**
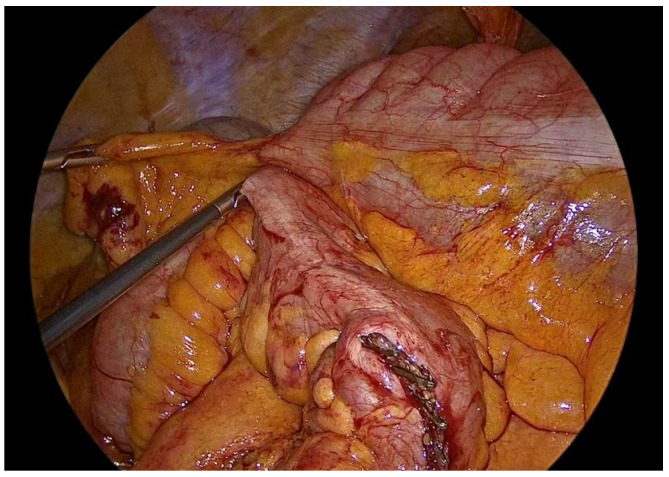
Alignment and positioning of the terminal ileum and transverse colon in an isoperistaltic, side-to-side configuration.

The transverse colon and terminal ileum are then aligned in an isoperistaltic, side-to-side orientation (Figure 6), at a distance of approximately 5 cm proximal to the stapled ends and secured in position using a barbed 3-0 suture measuring 15 cm in length. A single-layer handsewn intracorporeal anastomosis is subsequently fashioned using a Vicryl 3-0 suture measuring 34 cm in length. A barbed suture is preferred for bowel approximation, as it facilitates stable tissue apposition without the need for knot tying and helps maintain consistent tension along the anastomotic line. The Vicryl 3-0 suture is selected for anastomotic construction due to its favorable handling characteristics and ease of intracorporeal manipulation, allowing for precise and controlled suturing.

**Figure 7:**
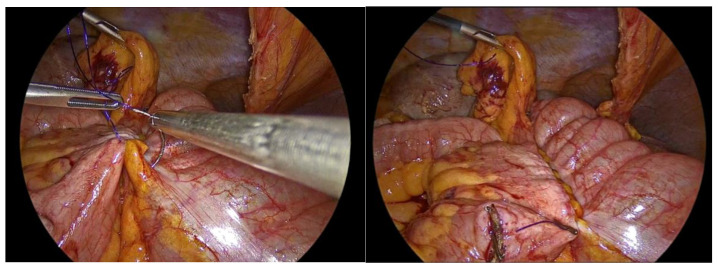
Side to side isoperistaltic sutured alignment of transverse colon with ileum.

The anastomosis is initiated with a continuous seromuscular layer using a barbed 3-0 non-absorbable suture, extending for approximately 8 to 10 cm along the posterior aspect, thereby providing structural reinforcement and support to the anastomotic construct (Figure 7).

**Figure 8:**
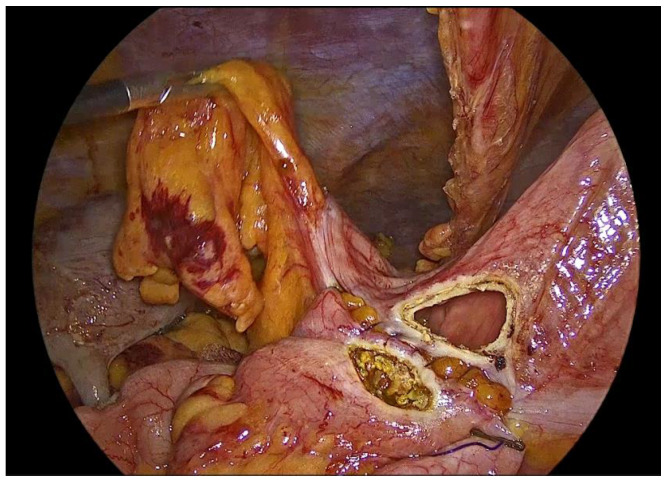
Enterotomies of bowel ends.

Enterotomies are subsequently created on the antimesenteric borders of both bowel segments, at a distance of approximately 1 cm from the outer suture line, with each enterotomy measuring 3 to 4 cm in length (Figure 8).

**Figure 9:**
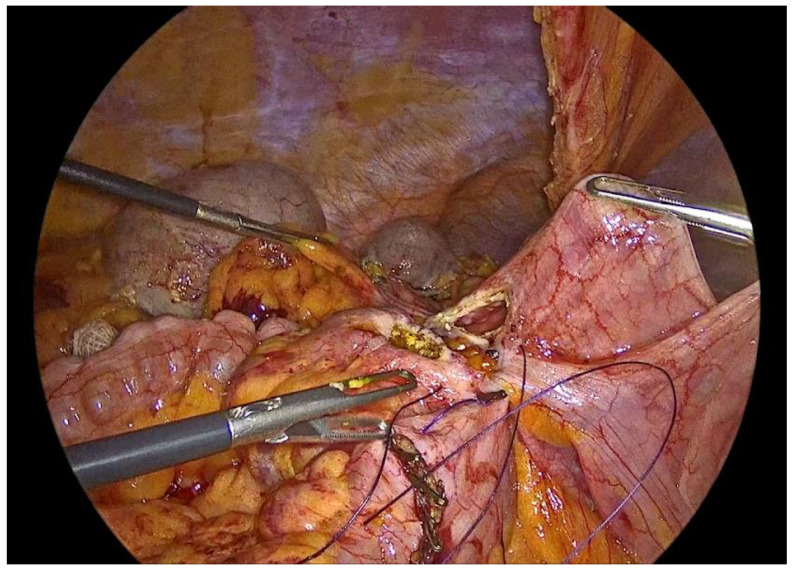
First suture of the anastomosis, using an absorbable 3-0 Vicryl Plus suture.

**Figure 10:**
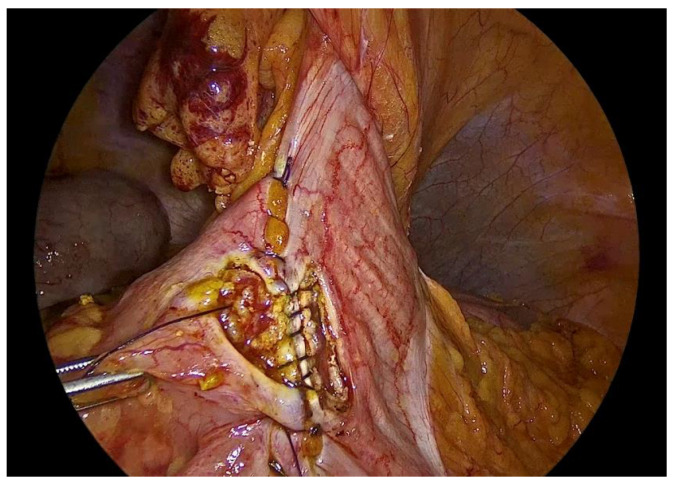
The inner layer of the anastomosis.

**Figure 11:**
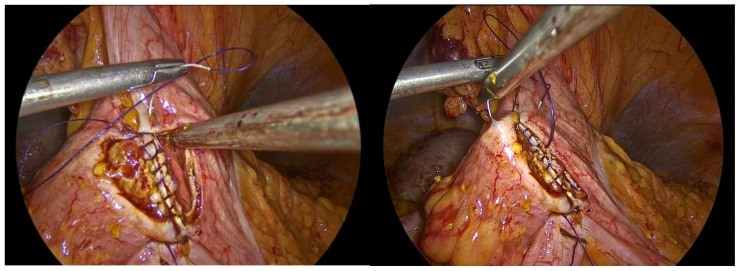
Completion of the inner full-thickness layer of the anastomosis and transition to construction of the anterior layer.

**Figure 12:**
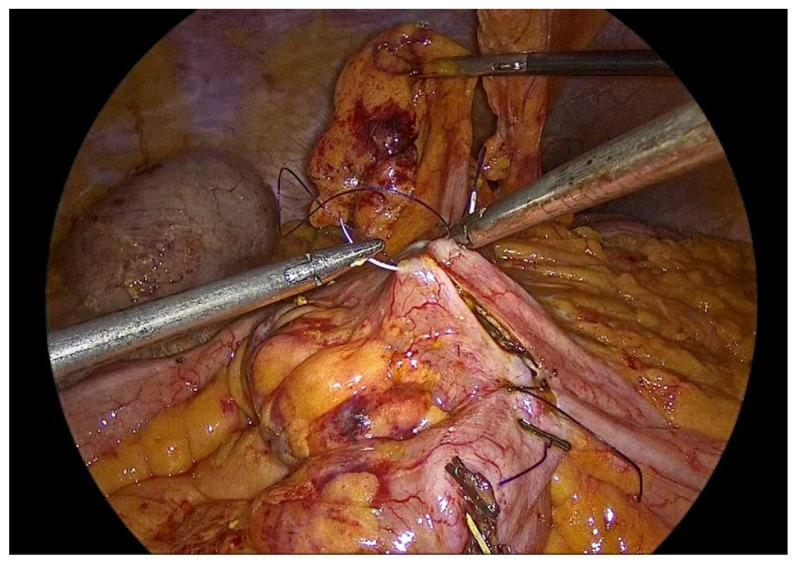
Anterior outer layer of the anastomosis.

**Figure 13:**
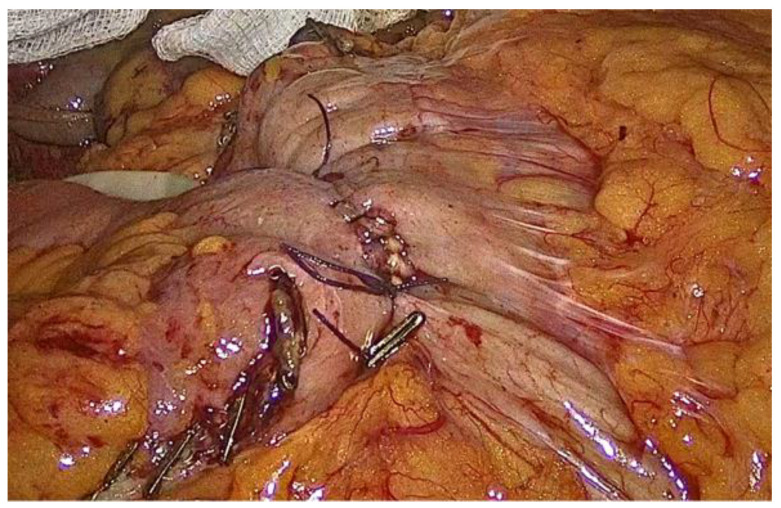
The completed single-layer handsewn intracorporeal anastomosis.

The inner layer of the anastomosis is constructed utilizing a continuous full-thickness absorbable 3-0 Vicryl Plus antibacterial suture, initiated at the proximal aspect relative to the surgeon’s position. Suturing proceeds from the proximal end toward the antimesenteric border, incorporating all layers of the bowel wall, with suture bites placed at regular intervals of 0.5 to 1 cm. Upon reaching the distal extremity of the enterotomy, the suture is continued along the opposite (anterior) side, and the anterior layer of the anastomosis is completed upon returning to the proximal starting point, where the suture is tied and secured to the initial knot (Figure 9, Figure 10, Figure 11, Figure 12 and Figure 13). 

To facilitate the safe and technically precise construction of the anastomosis, we systematically employ a combination of right-hand, left-hand, forehand, and backhand suturing techniques. The deliberate alternation between these approaches allows for optimal needle angulation, improved visualization of the operative field, and consistent suture placement along the entire circumference of the bowel. This technical versatility proves to be important, in situations where ergonomics and controlled movements directly influence the accuracy and integrity of the anastomosis.

During suturing, particular attention is directed toward proper tissue handling. We carefully avoid eversion of the colonic or ileal mucosa. Instead, we aim to achieve gentle inversion of both mucosal edges into the suture line. This inverted configuration promotes optimal serosa-to-serosa apposition aiming to reduce the risk of leakage. Equal emphasis is placed on uniform suture spacing, appropriate bite depth and consistent tension distribution to prevent tissue ischemia or tearing. Every effort is made to construct a symmetric, well-aligned anastomosis with a tension-free configuration and adequate luminal diameter.

Upon completion, the anastomosis is meticulously inspected. The entire suture line is carefully examined for gaps, bleeding points, or irregularities and the stapled bowel ends are assessed to confirm intact staple lines. Special attention is given to ensure adequate hemostasis and verifying the absence of hematoma formation or compromised perfusion.

Specimen extraction is carried out through a Pfannenstiel incision, with the routine use of a wound protector to ensure safe retrieval and to prevent contamination of the incision site. This approach is deliberately chosen in order to reduce surgical site infection rates, minimize postoperative pain, and decrease the risk of incisional hernia formation. By utilizing a low transverse suprapubic incision combined with protective measures, tissue trauma is limited, cosmetic outcomes are improved and overall postoperative morbidity is reduced.

The procedure is concluded laparoscopically with placement of a Penrose drain, positioned posterior to the anastomosis. This represents standard practice at our institution and is intended to minimize the risk of postoperative intra-abdominal fluid collection, particularly following extensive lymphadenectomy as performed during right colectomy. A final inspection of the abdominal cavity is performed to confirm hemostasis, after which pneumoperitoneum is released.

## 3. Clinical Experience

In our institutional practice, patients undergoing laparoscopic right colectomy follow a standardized preoperative preparation protocol. Mechanical bowel preparation was performed using oral polyethylene glycol solution the day prior to surgery. In addition, intravenous antibiotic prophylaxis was administered approximately one hour prior to skin incision, in strict accordance with institutional guidelines and colorectal surgery protocols. 

The mean operative time was approximately 200 min, a duration that is comparable to operative times reported for intracorporeal stapled anastomosis during the early and intermediate phases of the learning curve [20,21,22]. It is noteworthy that operative time for handsewn intracorporeal anastomosis is anticipated to be longer than that of stapled techniques; however, this differential diminishes progressively as cumulative surgical experience increases and the learning curve is traversed. In the present experience, the operating surgeon had already acquired proficiency in intracorporeal handsewn anastomosis through extensive prior experience in laparoscopic bariatric surgery, where intracorporeal handsewn gastrojejunal anastomosis is routinely performed during Roux-en-Y gastric bypass procedures. The learning curve for laparoscopic Roux-en-Y handsewn anastomosis was estimated at approximately 50 procedures, after which the technique was progressively adapted and applied to laparoscopic right colectomy. Importantly, the cases reported in the present study represent the period following standardization of the technique, thereby reflecting a mature and reproducible operative workflow. Over the course of this series, a measurable reduction in operative time was observed: total operative duration decreased from approximately 240 min during the initial procedures to 180 min in the most recent cases, while the time required specifically for anastomotic construction decreased from 40 min to 20 min. These findings suggest that, with adequate training and procedural standardization, the operative time differential between handsewn and stapled approaches can be substantially attenuated.

Estimated intraoperative blood loss was minimal across all procedures, ranging from approximately 20 to 50 mL. Of particular significance, no bleeding from the anastomotic line or hematoma formation was observed intraoperatively—a finding of considerable clinical relevance given the higher rates of anastomotic bleeding reported with stapled techniques in both laparoscopic and conventional open colorectal surgery [23,24,25]. Furthermore, the exclusive use of suture material for anastomotic construction, instead of the additional two to three endoscopic stapler cartridges typically required for intracorporeal stapled anastomosis, contributed to a measurable reduction in per-procedure disposable device utilization. This reduction translates into a meaningful decrease in overall procedural expenditure, thereby providing a substantial cost advantage of the handsewn technique.

Postoperatively, an enhanced recovery pathway was followed. Patients commenced a liquid diet on the first postoperative day, with gradual advancement to a soft and subsequently regular diet based on the return of bowel function and in the absence of nausea, vomiting, or abdominal distention. The time to first passage of flatus ranged from the second to the third postoperative day, consistent with the accelerated postoperative recovery commonly reported following intracorporeal anastomosis when compared with extracorporeal approaches [12,26,27]. The mean length of hospital stay ranged from three to four days, which is comparable to, and in certain instances shorter than, hospital stays reported for intracorporeal stapled anastomosis in laparoscopic right colectomy series [21]. Postoperative pain was limited, with minimal requirement for opioid analgesics, reflecting the reduced surgical trauma inherently associated with totally laparoscopic techniques and the utilization of a Pfannenstiel extraction incision [3].

This handsewn intracorporeal anastomotic technique has been routinely implemented in laparoscopic right colectomies, predominantly for malignant disease, over the past three years (2022–2025). A total of 68 consecutive procedures were completed laparoscopically using this standardized method, all performed by the same surgeon, thereby ensuring procedural consistency and technical uniformity. Postoperative complications were infrequent and of low severity, with no major complications (Clavien–Dindo III or above) observed. Notably, no anastomotic leaks were observed; anastomotic bleeding, stenosis, or hematoma formation were not identified in any patient, either during the immediate postoperative period or throughout the follow-up interval extending to 12 months. These outcomes are comparable to, and in certain parameters favorable when contrasted with, those reported for intracorporeal stapled anastomosis in the published literature and suggest that a handsewn approach does not compromise anastomotic safety when performed by surgeons with advanced laparoscopic skills and adequate experience [20,21].

All patients follow a standardized postoperative surveillance protocol. Following discharge, patients are reviewed at 10 days, 1 month, 6 months, and 12 months postoperatively. At each visit, evaluation encompasses wound assessment, nutritional status, and bowel function, with particular attention directed toward the early detection of symptoms suggestive of anastomotic stricture, including progressive changes in bowel habit, abdominal distention, and obstructive symptomatology.

Oncologic outcomes were satisfactory and aligned with established oncologic principles. Among 62 patients operated on for malignant disease, all resections achieved negative margins (R0) on final pathology. The median lymph node yield was 25 (range 14–42), and the pathologic stage distribution reflected a typical spectrum of disease. Oncological surveillance was conducted in accordance with established guidelines and in collaboration with oncologists. Adequate proximal and distal resection margins were achieved in all malignant cases, and lymph node harvest met accepted oncologic standards, confirming appropriate oncologic radicality. The use of a handsewn intracorporeal anastomosis did not adversely affect specimen quality, margin status, or nodal yield. Taken together, these findings support the feasibility, safety, and oncological adequacy of handsewn intracorporeal anastomosis and provide a robust clinical framework for its comparison with stapled techniques, as discussed in the subsequent sections.

## 4. Discussion

The concept of the ideal anastomosis in colorectal surgery has traditionally been defined by fundamental surgical principles, namely a technique that is safe, reproducible, and technically accessible, while minimizing postoperative morbidity and complication rates. Ideally, such an anastomosis should be amenable to performance by surgeons with varying levels of expertise, should not necessitate highly specialized or advanced technical skills, and should demonstrate consistently low rates of anastomotic failure across diverse clinical settings [28]. Despite significant advances in surgical technique, instrumentation, and perioperative care, no single anastomotic method has yet fulfilled all of these criteria. Consequently, there remains no universally accepted gold standard for anastomotic construction in colorectal surgery, and the optimal approach continues to be a subject of active debate and ongoing investigation. 

As previously discussed, numerous studies have systematically compared extracorporeal anastomosis (EA) with intracorporeal anastomosis (IA) in colorectal surgery, particularly in the setting of laparoscopic right colectomy. The preponderance of available evidence supports the superiority of IA over EA across several operative and postoperative parameters. Operative outcomes such as conversion rates to open surgery, incision length, and estimated blood loss have consistently been shown to be significantly lower in IA cohorts, provided that procedures are performed by experienced surgeons with advanced laparoscopic proficiency [10,11]. These findings are further corroborated by the IEA randomized clinical trial (Bollo et al., 2020), which demonstrated that intracorporeal anastomosis was associated with significantly faster recovery of digestive function (2.3 versus 3.3 days), reduced paralytic ileus rates (13% versus 30%), shorter incision length (6.7 versus 8.7 cm), and less postoperative pain in a cohort of 140 randomized patients [29]. The reduction in estimated blood loss is of particular clinical relevance, as it is directly associated with improved short-term outcomes and diminished postoperative morbidity [10,11,30].

Furthermore, the intracorporeal approach minimizes excessive bowel manipulation and mesenteric traction—maneuvers that are frequently unavoidable during extracorporeal anastomosis. By obviating the need for exteriorization of the bowel through a mini-laparotomy, IA substantially reduces the risk of mesenteric stretching, vascular compromise, hemorrhage, hematoma formation, and devascularization of the bowel ends, all of which may adversely impact anastomotic integrity and subsequent healing. These advantages likely contribute to the improved anastomotic viability observed in IA cohorts and support the increasing adoption of intracorporeal techniques in contemporary minimally invasive colorectal surgery [10,31,32].

Another well-documented benefit of intracorporeal anastomosis is the reduction in postoperative infectious complications, particularly surgical site infections. Smaller extraction incisions, diminished tissue trauma, and the avoidance of direct exposure of the wound to bowel contents collectively translate into lower wound infection rates and a decreased need for wound-related reintervention [10,11]. This advantage is particularly relevant in high-risk patient populations, including obese patients and those with multiple comorbidities, in whom wound-related complications are more frequent and associated with prolonged hospital stay and increased healthcare expenditure [33].

When evaluating the role of intracorporeal anastomosis across different patient risk profiles, studies have demonstrated that totally laparoscopic colectomies yield comparable surgical outcomes in high-risk patients when compared with low-risk populations [33]. Importantly, rates of anastomotic leakage, internal hernia formation, reoperation, and overall mortality do not differ significantly between IA and EA groups [10,31]. These findings suggest that IA can be safely applied across a broad spectrum of patients when appropriate surgical expertise is available. 

A noteworthy observation in the literature is that progression through the learning curve for intracorporeal anastomosis does not appear to adversely affect anastomotic leakage rates. On the contrary, perioperative complication rates are frequently lower in IA groups compared with EA cohorts, even during the early adoption phase [10]. This observation underscores the inherent technical advantages of the intracorporeal approach itself and suggests that once basic proficiency is achieved, intracorporeal anastomosis may offer more consistent and predictable outcomes than extracorporeal approaches.

Postoperative bowel function represents another important outcome parameter that consistently favors intracorporeal anastomosis. Multiple studies, including randomized controlled trials, have reported faster recovery of bowel movements, earlier tolerance of oral intake, and reduced incidence of postoperative ileus in IA patients [12,26,27,29]. These benefits are likely multifactorial, reflecting reduced bowel manipulation, a diminished systemic surgical stress response, and improved anastomotic geometry [4]. In particular, IA facilitates the construction of a tension-free anastomosis without the necessity for extensive mobilization of the transverse colon to accommodate exteriorization through a mini-laparotomy, thereby preserving the natural anatomical orientation of the bowel [4,31,34]. Despite these well-documented advantages, widespread adoption of IA remains limited, largely attributable to the advanced laparoscopic skills and manual dexterity required for intracorporeal anastomotic construction.

To date, and to the best of our knowledge, there exists a notable paucity of studies directly comparing intracorporeal handsewn and stapled anastomosis specifically within the context of laparoscopic right colectomies. In contrast, such comparisons have been more extensively explored in upper gastrointestinal surgery, where intracorporeal handsewn anastomosis has been routinely performed for decades [35], and more recently in the rapidly expanding field of robotic-assisted colorectal surgery [20,21,36,37]. 

The relative scarcity of laparoscopic data in this regard may be partially attributable to the inherent technical advantages offered by robotic surgical platforms, including three-dimensional high-definition visualization, articulated instruments that replicate wrist-like movements with seven degrees of freedom, enhanced ergonomics, and integrated tremor filtration systems. These technological features substantially lower the technical barriers to performing intracorporeal handsewn anastomosis and may account for the earlier and more widespread adoption of this technique in the robotic surgical paradigm [21,38].

Evidence derived from robotic studies suggests that intracorporeal handsewn anastomosis may be associated with increased operative time but a shorter overall length of hospital stay [21]. The prolonged operative duration is likely attributable to the inherent technical demands of intracorporeal suturing. However, this difference appears to diminish progressively with increasing surgeon experience and procedural volume [20]. Additionally, standardization of the individual steps involved in the construction of a handsewn anastomosis has been demonstrated to significantly reduce operative time, lending support to the notion that reproducibility and efficiency improve substantially with protocol-driven and standardized approaches [34].

Economic considerations constitute an additional dimension supporting the potential role of handsewn intracorporeal anastomosis. In standard intracorporeal stapled ileocolic anastomosis, a minimum of four to five firings of an endoscopic linear stapler are typically required: one each for transection of the terminal ileum and transverse colon, one for the creation of the side-to-side anastomosis, and one or two for closure of the common enterotomy [31]. The cost analysis of the Torino randomized trial demonstrated that surgical instrument costs were approximately €542 higher per patient in the intracorporeal stapled group compared with the extracorporeal group, a difference driven predominantly by the additional cartridge firings required for intracorporeal anastomotic construction [39]. By contrast, handsewn intracorporeal anastomosis eliminates the need for stapler firings for anastomosis construction and enterotomy closure, instead utilizing conventional suture material. In our approach, a barbed 3-0 suture and a single Vicryl 3-0 suture are used, the combined cost of which constitutes only a minor fraction of the cost of the corresponding stapler cartridges. In our institutional experience, stapler use was limited to bowel transection, thereby avoiding two to three cartridge firings per procedure and resulting in a meaningful reduction in per-case expenditure.

Bleeding from the anastomotic line represents another critical determinant of anastomotic success and postoperative morbidity. Several studies have demonstrated that anastomotic bleeding occurs more frequently in stapled anastomoses and, although uncommon, can occasionally necessitate endoscopic or surgical intervention [23]. Concordant findings have been reported in conventional open colorectal surgery, where stapled anastomoses have been associated with higher rates of postoperative bleeding and anastomotic stricture formation compared with handsewn techniques [24,25]. These observations collectively suggest that handsewn anastomosis may afford superior control over tissue approximation and hemostasis, potentially translating into improved anastomotic quality and a reduced need for postoperative intervention.

### Limitations

Several limitations of the present study merit acknowledgment. First, the retrospective and single-center nature of the study, with all procedures performed by a single surgeon, limits the generalizability of the findings to other surgical settings and operators. Although the single-surgeon design ensures technical consistency, it also introduces potential selection bias and precludes assessment of the technique’s reproducibility across surgeons with varying levels of laparoscopic expertise. The institutional experience described reflects a single-surgeon practice and is presented for illustrative purposes only. Second, the absence of a direct comparator group, such as patients undergoing intracorporeal stapled anastomosis, precludes definitive conclusions regarding the relative superiority of the handsewn technique. Third, the sample size of 68 patients, while adequate to demonstrate feasibility and safety, may be insufficient to detect uncommon complications such as late anastomotic stricture or long-term oncological outcomes. Fourth, as this manuscript is intended as a descriptive presentation of the surgical technique, detailed patient demographics, comorbidity profiles, and tumor staging characteristics are not included in this report. Future prospective, multicenter, and ideally randomized comparative studies with larger sample sizes and extended follow-up periods are warranted to validate these preliminary findings and to more precisely define the role of handsewn intracorporeal anastomosis in laparoscopic colorectal surgery.

## 5. Conclusions

In this study, we present a detailed and standardized technique for performing an isoperistaltic side-to-side handsewn intracorporeal ileocolic anastomosis following laparoscopic right colectomy. Our institutional experience, encompassing 68 consecutive procedures with no anastomotic leaks or anastomosis-related complications, demonstrates that this technique can be safely and effectively executed in a fully intracorporeal manner for both malignant and benign conditions necessitating right colectomy or ileocecal resection. When fundamental surgical principles, including meticulous technique, adequate blood supply, and tension-free anastomotic construction, are meticulously observed, intracorporeal handsewn anastomosis represents a feasible, safe, and cost-effective alternative to stapled techniques. Moreover, the elimination of mechanical stapling devices confers a notable economic advantage without compromising anastomotic quality. Further prospective, comparative, and multicenter studies are needed to validate these findings and to establish the broader applicability and long-term outcomes of this technique in the evolving landscape of minimally invasive colorectal surgery.

## Data Availability

The original contributions presented in this study are included in the article. Further inquiries can be directed to the corresponding author.

## Abbreviations

The following abbreviations are used in this manuscript:

IA	Intracorporeal Anastomosis
EA	Extracorporeal Anastomosis
